# Supplementary material for: Estimation of true dates of various flowering stages at a centennial scale by applying a Bayesian statistical state space model
Source: PLoS One. 2025 Feb 5;20(2):e0317708. doi: 10.1371/journal.pone.0317708 (PMC11798523; doi:10.1371/journal.pone.0317708)
Supplement: S2 File — (DOCX) [file pone.0317708.s002.docx]

14 January 2025

Dear Dr. Bahram Heidari

Academic editor of *PLOS ONE*

Dear Dr. Heidari

We sincerely appreciate your coordination of the review and comments on the following submitted paper:

**ID:** PONE-D-24-36692

**Title:** Estimation of true dates of various flowering stages at a centennial scale by applying a Bayesian statistical state space model

PLOS ONE

We have substantially checked and revised the manuscript in accordance with the editorial comments. In addition, we have revised a minor point in lines 202−203. To use correctly terms, we have revised “confidence interval” into “credible interval” in the main text. The portions of the manuscript that we revised are coloured red. Our detailed responses to the comments are provided in this letter. We hope that our responses and the resulting changes will be satisfactory, but we will be happy to work with you to resolve any remaining issues.

Sincerely yours,

Shin Nagai (on behalf of all authors)

Shin Nagai, Ph.D.

Corresponding Author

Senior researcher

Research Institute for Global Change

Japan Agency for Marine-Earth Science and Technology (JAMSTEC)

Email: [nagais@jamstec.go.jp](mailto:nagais@jamstec.go.jp), [pjn00210@biglobe.ne.jp](mailto:pjn00210@biglobe.ne.jp)

Phone: +81-45-778-5594

Address: 3173-25 Showa-machi, Kanazawa-ku, Yokohama 236-0001, Japan

**Journal requirements:**

*Requirement 1:*

Please ensure that the author list and affiliations are correct on the title page of your manuscript, and that your author contributions, competing interests, and financial disclosure are correct as listed below. All of these sections will be indexed in PubMed and published by PLOS ONE as you have written them. Please email plosone@plos.org if any changes to this content need to be made.

Shin Nagai:

Conceptualization

Data curation

Formal analysis

Investigation

Methodology

Validation

Visualization

Writing – original draft

Writing – review & editing

Hakuryu Fujiwara:

Data curation

Investigation

Shinjiro Sugiyama:

Data curation

Investigation

Hiroshi Morimoto:

Formal analysis

Methodology

Validation

Writing – review & editing

Taku M. Saitoh:

Validation

Writing – review & editing

Please see here for the full list and definition of contributor roles: http://journals.plos.org/plosone/s/authorship#loc-author-contributions

*Answer:*

We have confirmed.

Please ensure that the Competing Interests and Financial Disclosure statements listed below are suitable for publication. These sections will be indexed in PubMed and published by PLOS ONE as you have written them. Please email plosone@plos.org if any changes to these statements need to be made.

Competing Interests:

The authors have declared that no competing interests exist.

Financial Disclosure:

NS was supported by a KAKENHI grant (JSPS 21H05178) from the Japan Society for the Promotion of Science.

https://www.jsps.go.jp/english/e-grants/index.html

This grant supported our activities regarding data collection and preparation of the manuscript.

*Answer:*

We have confirmed. Could you please revise as follows? We guess one of grand number missed in the revision process.

NS was supported by a KAKENHI grant (JSPS 21H05178 and JP24K21357) from the Japan Society for the Promotion of Science.

*Requirement 2:*

There is a difference between the author names listed in the submission and your manuscript file.

An author is listed as Nagai Shin in the manuscript file but as Shin Nagai in the online submission form. If the name is incorrect in the manuscript, please correct this. If the name is incorrect in the submission form, please let us know so we can correct the author’s profile for you.

Please note that the use of middle initials/names should be consistent in the manuscript’s author list, the submission form, and the author contributions initials. Furthermore, author names in the byline in your manuscript should also include the author's first names before their surnames. The author names will be typeset exactly as they appear in the manuscript so please ensure that the information provided is correct.

*Answer:*

I want to show “Nagai Shin” on the publication. Therefore, could you please correct the author’s profile (first name and surname) on the online system?

*Requirement 3:*

There is a difference between the corresponding email listed in the submission and your manuscript file.

A corresponding email is listed as nagais@jamstec.go.jp in the manuscript file but as pjn00210@biglobe.ne.jp in the online submission form. If the email is incorrect in the manuscript, please correct this. If the email is incorrect in the submission form, please let us know so we can correct the author’s profile for you.

*Answer:*

[nagais@jamstec.go.jp](mailto:nagais@jamstec.go.jp) is my official email address. Therefore, could you please correct the author’s profile (contact email address) on the online system?

*Requirement 4:*

We note that you have included a Competing Interests Statement within the body of your manuscript. Please note that declarative statements should not be included within the manuscript. Please ensure that the statement provided in the submission system is correct, and then remove the statement from your manuscript file.

*Answer:*

We have deleted this part in the main manuscript.

*Requirement 5:*

We note that you have included the Author Contributions in your manuscript. Please note that author contributions should not be included in the manuscript.

*Answer:*

We have deleted this part in the main manuscript.

*Requirement 6:*

The number of Supporting Information captions in your manuscript does not match the number of Supporting Information files uploaded to Editorial Manager. Please ensure that the Supporting Information files you have uploaded match the number of captions in your Supporting Information Captions section.

A Supporting Information file containing multiple supporting figures, tables, etc. is acceptable; however, it must have just one caption in the Supporting Information Captions section of the manuscript.

Please see these examples for more guidance: <http://journals.plos.org/plosone/s/file?id=wjVg/PLOSOne_formatting_sample_main_body.pdf>

*Answer:*

We have added the following Supplementary Information Captions section. In accordance with this revision, we have revised the main text in line 120 and the file name of supplementary file.

**Supplementary Information Captions section**

Supplementary file 1. Observation records, which were converted to the day of year (DOY) for analysis, in literature, photographs, and the private collection of Hakuryu Fujiwara (Supplementary_file_1.csv).

*Requirement 7:*

To prevent production delays, we recommend using the Author Formatting Checklist to confirm that your paper meets PLOS ONE’s typesetting requirements for References, Tables, and Figures: http://journals.plos.org/plosone/s/file?id=c819/plos-one-author-formatting-checklist.docx.

This checklist is a reference tool for you; please do not upload the completed Author Formatting Checklist with your submission files.

*Answer:*

We have revised the format of manuscript in accordance with the guideline.

*Requirement 8:*

To ensure your figures meet our technical requirements, please run each figure included in your submission files through the PACE tool: https://pacev2.apexcovantage.com/. PACE will assess whether your figures meet our technical requirements and will fix the figure(s) or identify any problem(s) that cannot be automatically fixed. It can also convert figures to TIFF format, resize, and rename figures to meet our naming conventions.

To use PACE, first register as a user. Follow the instructions on the site for assessing and converting your figure files. If you experience any difficulty using this tool or have questions about any of the figures and/or images in your paper, please inform the journal office in your response letter.

*Answer:*

We have run the PACE tool. We believe there is no significant issue.
